# Supplementary material for: Assisted Reproductive Technology Results Using Donor or Partner Sperm: A Danish Nationwide Register-Based Cohort Study
Source: J Clin Med. 2023 Mar 29;12(7):2571. doi: 10.3390/jcm12072571 (PMC10095571; doi:10.3390/jcm12072571)
Supplement: Supplementary file 1 [file jcm-12-02571-s001.zip › jcm-2235061-supplementary.pdf]

Supplementary Materials

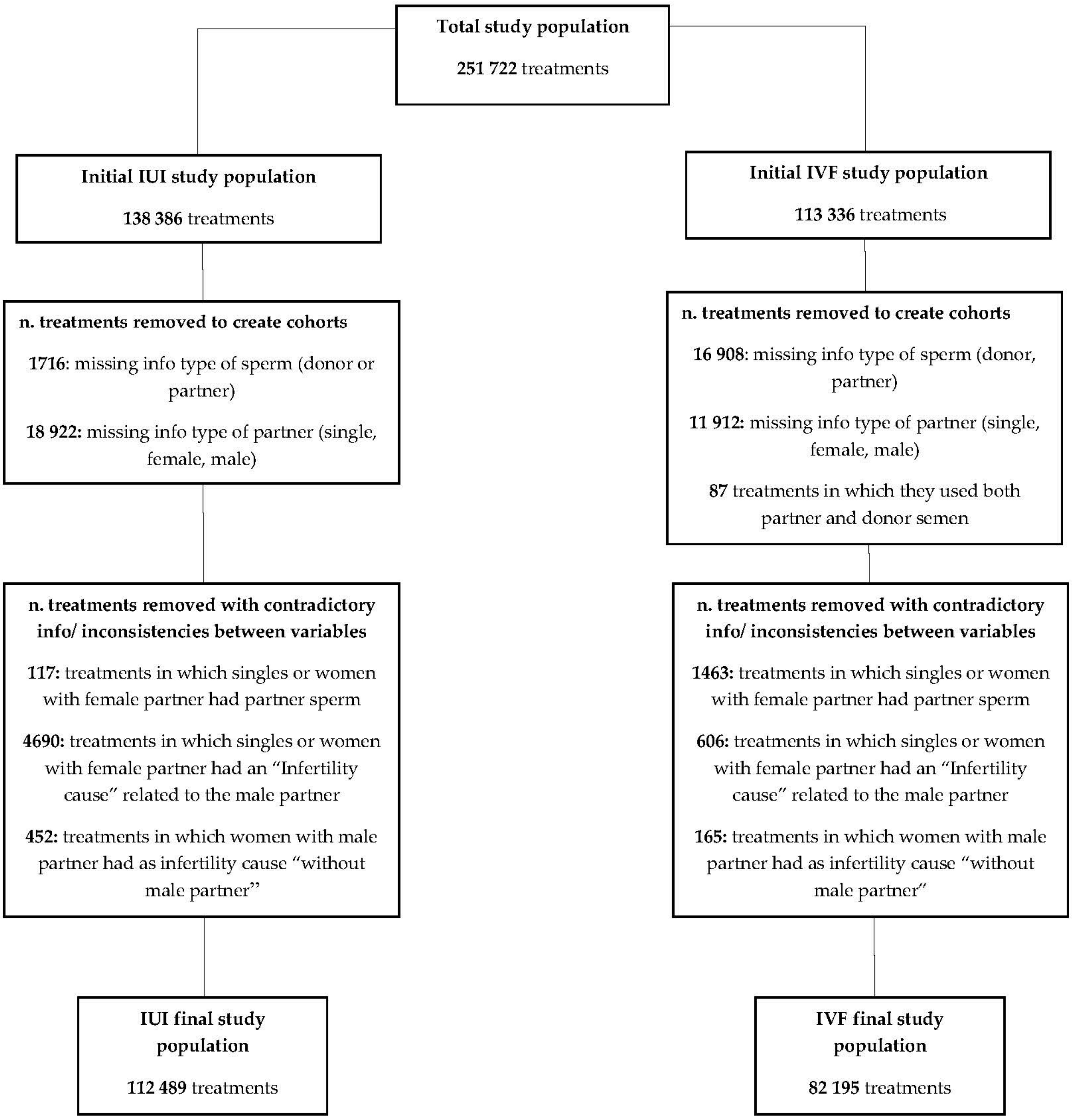

Figure S1. Flowchart illustrating the patient selection process.

Table S1. Sensitivity analyses performed by including one covariate at a time to assess the individual effect on the odds ratio (OR) result.

IUI Treatments			
**Unexposed Cohort**	**Exposed Cohorts**		
**Treatments in women with male partners using partner semen**	**Treatments in women with male partners using donor sperm**	**Treatments in single women using donor sperm**	**Treatments in women with female partners using donor sperm**
**Crude OR (95% CI)**	1.43 (1.34–1.52)	0.94 (0.90–0.99)	1.52 (1.40–1.66)
**Single-covariate-adjusted OR (95% CI)**			
Age at treatment	1.50 (1.41–1.60)	1.21 (1.14–1.27)	1.49 (1.37–1.63)
No. of treatments	1.40 (1.31–1.51)	0.97 (0.92–1.02)	1.58 (1.46–1.72)
BMI	1.40 (1.31–1.51)	0.93 (88–0.98)	1.48 (1.34–1.63)
Smoking at time of treatment	1.38 (1.28–1.48)	0.94 (0.89–1.00)	1.50 (1.36–1.65)
Alcohol consumed	1.38 (1.29–1.48)	0.94 (0.89–1.00)	1.49 (1.35–1.65)
Calendar year of treatment	1.43 (1.34–1.52)	0.94 (0.90–0.99)	1.53 (1.40–1.66)
Infertility diagnosis	1.43 (1.33–1.53)	1.08 (0.99–1.18)	1.75 (1.57–1.96)
**IVF Treatments**			
**Treatments in women with male partners using partner sperm**	**Treatments in women with male partners using donor sperm**	**Treatments in single women using donor sperm**	**Treatments in women with female partners using donor sperm**
**Crude OR (95% CI)**	1.03 (0.92–1.16)	0.60 (0.55-0.65)	1.16 (1.00-1.36)
**Single-covariate-adjusted OR (95% CI)**			
Age at treatment	1.13 (1.01–1.27)	0.92 (0.85–0.99)	1.24 (1.06–1.45)
No. of treatments	1.07 (0.96–1.19)	0.60 (0.56–0.65)	1.15 (0.99–1.34)
BMI	1.05 (0.92–1.18)	0.60 (0.55–0.66)	1.16 (0.97–1.39)
Smoking at time of treatment	1.04 (0.91–1.17)	0.60 (0.55–0.67)	1.22 (1.02–1.45)
Alcohol consumed	1.04 (0.92–1.18)	0.60 (0.55–0.66)	1.21 (1.02–1.45)
Calendar year of treatment	1.04 (0.92–1.16)	0.59 (0.55–0.64)	1.15 (0.99–1.35)
Type of treatment (fresh/frozen)	1.04 (0.93–1.16)	0.59 (0.55–0.64)	1.17 (1.00–1.37)
Infertility diagnosis	0.99 (0.88–1.11)	0.67 (0.59–0.77)	1.30 (1.07–1.57)

Table S2. Sensitivity analysis that included female infertility diagnosis.

IUI Treatments		
**Exposed Cohorts**		**Live Births**
**Treatments in women with male partners using donor sperm**	**Crude OR (95% CI)**	1.43 (1.34–1.52)
	**Adjusted OR [a] (95% CI)**	1.46 (1.35–1.59)
**Treatments in single women using donor sperm**	**Crude OR (95% CI)**	0.94 (0.90–0.99)
	**Adjusted OR [a] (95% CI)**	1.36 (1.24–1.50)
**Treatments in women with female partners using donor sperm**	**Crude OR (95% CI)**	1.52 (1.40–1.66)
	**Adjusted OR [a] (95% CI)**	1.72 (1.51–1.95)
**IVF Treatments**		
**Exposed Cohorts**		**Live Births**
**Treatments in women with male partners using donor sperm**	**Crude OR (95% CI)**	1.03 (0.92–1.16)
	**Adjusted OR [b] (95% CI)**	1.14 (1.00–1.29)
**Treatments in single women using donor sperm**	**Crude OR (95% CI)**	0.60 (0.55–0.65)
	**Adjusted OR [b] (95% CI)**	0.97 (0.85–1.12)
**Treatments in women with female partners using donor sperm**	**Crude OR (95% CI)**	1.16 (1.00–1.36)
	**Adjusted OR [b] (95% CI)**	1.37 (1.11–1.69)

a: adjusted for age at treatment, calendar year of treatment, BMI, smoking at the time of treatment, alcohol consumed, no. of treatments, and infertility diagnosis. b: adjusted for age at treatment, calendar year of treatment, BMI, smoking at the time of treatment, alcohol consumed, type of treatment, no. of treatments, and infertility diagnosis. The unexposed cohort used as a reference was treatments in women with male partners using partner sperm.

Table S3. Overview of male infertility diagnoses.

IUI Treatments				
	Exposed Cohort			Unexposed Cohort
**Male infertility diagnosis, n (%)**	**Women with a male partner using donor semen**	**Single women using donor semen**	**Women with a female partner using donor semen**	**Women with a male partner using partner semen**
**Aspermia**	440 (5.01)			46 (0.06)
**Azoospermia**	3291 (37.48)			345 (0.43)
**Oligozoospermia**	1036 (11.80)			7607 (9.40)
**Oligoteratozoospermia**	1035 (11.79)			6333 (7.82)
**Other reasons** [a]	225 (2.56)			982 (1.21))
**Unexplained**	1359 (15.48)			33,286 (41.12)
**Retrograde ejaculation**	14 (0.16)			36 (0.04)
**Normal quality**	1380 (15.72)			32,314 (39.92)
**IVF Treatments**				
	Exposed Cohort			Unexposed Cohort
**Male infertility diagnosis, n (%)**	**Women with a male partner using donor semen**	**Single women using donor semen**	**Women with a female partner using donor semen**	**Women with a male partner using partner semen**
**Aspermia**	109 (5.63)			120 (0.16)
**Azoospermia**	766 (39.55)			1556 (2.09)
**Oligozoospermia**	307 (15.85)			16,824 (22.61)
**Oligoteratozoospermia**	240 (12.39)			12,034 (16.17)
**Other reasons** [a]	61 (3.15)			1391 (1.87)
**Unexplained**	246 (12.70)			17,889 (24.04)
**Retrograde ejaculation**	9 (0.46)			45 (0.06)
**Normal quality**	199 (19.27)			24,566 (33.01)

a: other reasons included a diagnosis of previous sterility, infection with HIV, and missing data.

Table S4. Sensitivity analyses that included only male partners of women using partner sperm with good sperm quality or reduced sperm quality.

Good Sperm Quality IUI Treatments		
**Exposed Cohorts**		**Live Births**
**Treatments in women with male partners using donor sperm**	**Yes, n (%)**	1461 (16.64)
	**No, n (%)**	7319 (83.36)
	**Crude OR (95% CI)**	1.4 (1.3–1.50)
	**Adjusted OR** [a] **(95% CI)**	1.57 (1.45–1.70)
**Treatments in single women using donor sperm**	**Yes, n (%)**	2131 (11.66)
	**No, n (%)**	16 152 (88.34)
	**Crude OR (95% CI)**	0.92 (0.87–0.98)
	**Adjusted OR** [a] **(95% CI)**	1.25 (1.17–1.35)
**Treatments in women with female partners using donor sperm**	**Yes, n (%)**	787 (17.58)
	**No, n (%)**	3690 (82.42)

	Crude OR (95% CI)	1.49 (1.36–1.63)
	Adjusted OR [a] (95% CI)	1.49 (1.34–1.66)
Unexposed cohort		
Treatments in women with male partners using partner sperm	Yes, n (%)	4044 (12.51)
	No, n (%)	28 270 (87.49)
Good Sperm Quality IVF Treatments		
Exposed Cohorts		Live Births
Treatments in women with male partners using donor sperm	Yes, n (%)	510 (26.33)
	No, n (%)	1427 (73.67)
	Crude OR (95% CI)	1.09 (0.97–1.23)
	Adjusted OR [b] (95% CI)	1.21 (1.06–1.38)
Treatments in single women using donor sperm	Yes, n (%)	862 (17.18)
	No, n (%)	4155 (82.82)
	Crude OR (95% CI)	0.63 (0.58–0.69)
	Adjusted OR [b] (95% CI)	0.92 (0.83–1.01)
Treatments in women with female partners using donor sperm	Yes, n (%)	234 (28.68)
	No, n (%)	582 (71.32)
	Crude OR (95% CI)	1.23 (1.05–1.43)
	Adjusted OR [b] (95% CI)	1.25 (1.04–1.50)
Unexposed cohort		
Treatments in women with male partners using partner sperm	Yes, n (%)	6063 (24.68)
	No, n (%)	18 503 (75.32)
Reduced Sperm Quality IUI Treatments		
Exposed Cohorts		Live Births
Treatments in women with male partners using donor sperm	Yes, n (%)	1461 (16.64)
	No, n (%)	7319 (83.36)
	Crude OR (95% CI)	1.52 (1.41–1.65)
	Adjusted OR [a] (95% CI)	1.59 (1.45–1.74)
Treatments in single women using donor sperm	Yes, n (%)	2131 (11.66)
	No, n (%)	16 152 (88.34)
	Crude OR (95% CI)	
	Adjusted OR [a] (95% CI)	
Treatments in women with female partners using donor sperm	Yes, n (%)	787 (17.58)
	No, n (%)	3690 (82.42)
	Crude OR (95% CI)	1.01 (0.94–1.08)
	Adjusted OR [a] (95% CI)	1.29 (1.19–1.41)
Unexposed cohort		
Treatments in women with male partners using partner sperm	Yes, n (%)	1663 (11.58)
	No, n (%)	12704 (88.42)
Reduced Sperm Quality IVF Treatments		
Exposed Cohorts		Live Births
Treatments in women with male partners using donor sperm	Yes, n (%)	510 (26.33)
	No, n (%)	1427 (73.67)
	Crude OR (95% CI)	0.95 (0.84–1.06)
	Adjusted OR [b] (95% CI)	1.10 (0.96–1.25)
Treatments in single women using donor sperm	Yes, n (%)	862 (17.18)
	No, n (%)	4155 (82.82)
	Crude OR (95% CI)	0.55 (0.51–0.60)

	**Adjusted OR[b] (95% CI)**	0.810 (0.74–0.89)
**Treatments in women with female partners using donor sperm**	**Yes, n (%)**	234 (28.68)
	**No, n (%)**	582 (71.32)
	**Crude OR (95% CI)**	1.06 (0.91–1.25)
	**Adjusted OR [b] (95% CI)**	1.14 (0.95–1.37)
**Unexposed cohort**		
**Treatments in women with male partners using partner sperm**	**Yes, n (%)**	8589 (27.31)
	**No, n (%)**	22 861 (72.69)

a: adjusted for age at treatment, calendar year of treatment, BMI, smoking at the time of treatment, alcohol consumed, and no. of treatments. b: adjusted for age at treatment, calendar year of treatment, BMI, smoking at the time of treatment, alcohol consumed, type of treatment, and no. of treatments.

Table S5. Crude and adjusted odds ratios (ORs) for biochemical pregnancy and clinical pregnancy in women with male partners using donor sperm, single women using donor sperm, women with female partners using donor sperm, and women with male partners using partner sperm in IUI and IVF treatments.

**IUI Treatments**			
**Exposed Cohorts**		**Biochemical Pregnancy (hCG)**	**Clinical Pregnancy (Ultrasound)**
**Treatments in women with male partners using donor sperm (N = 8780)**	**Yes, n (%)**	1816 (20.7)	1609 (88.6)
	**No, n (%)**	6254 (71.2)	150 (8.3)
	**Missing, n (%)**	710 (8.1)	57 (3.1)
	**Crude OR (95% CI)**	1.39 (1.30–1.48)	1.25 (1.04–1.51)
	**Adjusted OR [a] (95% CI)**	1.42 (1.33–1.52)	1.32 (1.07–1.62)
**Treatments in single women using donor sperm (N = 18,283)**	**Yes, n (%)**	2902 (15.9)	2410 (83.0)
	**No, n (%)**	13 555 (74.1)	263 (9.1)
	**Missing, n (%)**	1826 (10.0)	229 (7.9)
	**Crude OR (95% CI)**	1.02 (0.97–1.07)	1.07 (0.92–1.25)
	**Adjusted OR [a] (95% CI)**	1.21 (1.14–1.29)	1.37 (1.15–1.64)
**Treatments in women with female partners using donor sperm (N = 4477)**	**Yes, n (%)**	1002 (22.4)	717 (71.6)
	**No, n (%)**	3236 (72.3)	90 (9.0)
	**Missing, n (%)**	239 (5.3)	195 (19.5)
	**Crude OR (95% CI)**	1.48 (1.36–1.60)	0.93 (0.73–1.18)
	**Adjusted OR [a] (95% CI)**	1.48 (1.35–1.63)	1.08 (0.81–1.45)
**Unexposed cohort**			
**Treatments in women with male partners using partner sperm (N = 80,949)**	**Yes, n (%)**	12,130 (15.0)	10,674 (88.0)
	**No, n (%)**	57 889 (71.5)	1247 (10.3)
	**Missing, n (%)**	10 930 (13.5)	209 (1.7)
**IVF Treatments**			
**Exposed Cohorts**		**Biochemical Pregnancy (hCG)**	**Clinical Pregnancy (Ultrasound)**
**Treatments in women with male partners using donor sperm (N = 1937)**	**Yes, n (%)**	792 (40.9)	647 (81.7)
	**No, n (%)**	1132 (58.4)	139 (17.6)
	**Missing, n (%)**	183 (0.7)	6 (0.8)
	**Crude OR (95% CI)**	1.15 (1.04–1.27)	0.87 (0.71–1.07)
	**Adjusted OR [b] (95% CI)**	1.26 (1.12–1.41)	0.90 (0.71–1.13)
**Treatments in single women using donor sperm (N = 5017)**	**Yes, n (%)**	1581 (31.5)	1218 (77.0)
	**No, n (%)**	3431 (68.4)	352 (22.3)
	**Missing, n (%)**	5 (0.1)	11 (0.7)
	**Crude OR (95% CI)**	0.76 (0.71–0.81)	0.65 (0.57–0.74)
	**Adjusted OR [b] (95% CI)**	0.99 (0.92–1.07)	0.79 (0.68–0.91)

**Treatments in women with female partners using donor sperm (N = 816)**	**Yes, n (%)**	344 (42.2)	288 (83.7)
	**No, n (%)**	471 (57.7)	54 (15.7)
	**Missing, n (%)**	1 (0.12)	2 (0.6)
	**Crude OR (95% CI)**	1.20 (1.03–1.40)	1.00 (0.72–1.39)
	**Adjusted OR [b] (95% CI)**	1.19 (1.00–1.43)	1.27 (0.86–1.87)
**Unexposed cohort**			
**Treatments in women with male partners using partner sperm (N = 74,425)**	**Yes, n (%)**	27,707 (37.2)	22,998 (83.0)
	**No, n (%)**	45,566 (61.2)	4316 (15.6)
	**Missing, n (%)**	1152 (1.6)	393 (1.4)

a: adjusted for age at treatment, calendar year of treatment, BMI, smoking at the time of treatment, alcohol consumed, and no. of treatments. b: adjusted for age at treatment, calendar year of treatment, BMI, smoking at the time of treatment, alcohol consumed, type of treatment, and no. of treatments. The analysis of the clinical pregnancy was based only on those women who had a positive biochemical pregnancy.
